# Editorial: Treatment and management of stimulant use disorder and co-occurring disorders

**DOI:** 10.3389/fpsyt.2026.1848661

**Published:** 2026-04-22

**Authors:** Jeremy Weleff, Mohit Singh, David Crockford

**Affiliations:** 1 Department of Psychiatry, University of Alberta, Edmonton, AB, Canada; 2 Department of Psychiatry, Yale School of Medicine, New Haven, CT, United States; 3 University of Calgary, Cumming School of Medicine, Department of Psychiatry, Calgary, AB, Canada; 4 Hotchkiss Brain Institute, University of Calgary, Calgary, AB, Canada

**Keywords:** cocaine, cocaine (crack), concurrent disorders, methamphetamine, stimulant use disorder, substance use disorders

Stimulant use disorders have emerged as an increasingly urgent clinical and public health challenge (1–3). Rising harms related to methamphetamine, cocaine, and other stimulants are unfolding within an increasingly toxic drug supply, often shaped by the growing presence of potent synthetic opioids and other novel psychoactive substances. Recent work has further shown the growing burden of co-occurring stimulant and fentanyl in drug checking samples, alongside evidence that absolute fentanyl, methamphetamine, and cocaine concentrations in clinical urine drug testing specimens increased substantially across the past decade in the United States (4, 5). Against this backdrop, stimulant use disorders rarely present in isolation and are frequently intertwined with broader structural and health-system vulnerabilities as well as often complicated psychiatric presentations including suicidality, aggression, psychosis, and attention-deficit/hyperactivity disorder (ADHD) (6–10). Despite this complexity, the evidence base guiding clinical management remains incomplete, particularly where co-occurring disorders are concerned.

The contributions assembled in this Research Topic reflect both the breadth of the problem and the plurality of responses required. Two opinion pieces frame the current moment with clarity. Mills et al. call for an urgent, multilevel response to the stimulant-fentanyl overdose crisis, emphasizing the need to pair evidence-based interventions with structurally informed and culturally responsive implementation strategies. Balasanova and Davids focus on methamphetamine-related harms in the Midwestern United States, showing how regional service gaps, policy barriers, and strained systems of care shape the lived reality of stimulant-related morbidity and mortality. Together, these papers remind us that stimulant use disorders are not only a matter of individual pathology, but also of policy, geography, and access.

Other contributions deepen the epidemiological and clinical picture. In a population-based study from British Columbia, Desai et al. report rising incident stimulant use disorder diagnoses following the onset of the COVID-19 public health emergency, underscoring the extent to which recent social disruption may have intensified stimulant-related harms. Adam et al., in a systematic review and meta-analysis, further highlight the substantial psychiatric burden associated with amphetamine-type stimulant use, including depression, hallucinations, and suicidality. Complementing this broader view, Yalçın and Paltun examine methamphetamine-related psychiatric emergencies in Türkiye, drawing attention to the acute clinical risks that often accompany stimulant use, including psychotic symptoms, self-harm, aggression, and suicidal ideation (Ergelen Yalçın and Paltun).

The Research Topic also points toward the importance of better tools and more tailored treatment frameworks. Chung et al. examine the diagnostic performance of the Chinese version of the Severity of Dependence Scale for DSM-5 stimulant use disorder, illustrating the value of context-sensitive measurement in both research and care (16). Oliva et al. outline a protocol for a systematic review and meta-analysis of pharmacotherapies for co-occurring stimulant use disorder and ADHD, a clinically important but still underdeveloped area of practice. At a more translational level, Barbosa-Mendez and Salazar-Juárez present preclinical findings on a cocaine vaccine, reflecting ongoing efforts to broaden the therapeutic horizon beyond currently available approaches.

The papers in this Research Topic highlight stimulant use disorder as a field requiring integrated attention across substances, psychiatric and medical comorbidities, levels of care, and research traditions spanning epidemiology to translational therapeutics. They suggest that progress will depend not only on identifying effective interventions, but also on developing approaches that are diagnostically precise, clinically realistic, and responsive to the structural and social contexts in which stimulant-related harms occur.

As stimulant-related morbidity and mortality continue to evolve, so too must the frameworks used to understand and treat them. We hope this Research Topic contributes to that effort by advancing a more clinically useful, and evidence-informed understanding of stimulant use disorders and their co-occurring disorders.
